# The forgotten stent: late complication in a patient with neobladder

**DOI:** 10.1100/tsw.2006.81

**Published:** 2006-03-30

**Authors:** Riccardo Autorino, Antonio Maschio, Umberto Pane, Marco De Sio, Luca Cosentino, Giuseppe Quarto, Salvatore Mordente, Ferdinando Di Giacomo, Luigi Santini, Massimo D'Armiento

**Affiliations:** ^1^ Clinica Urologica, Seconda Università degli Studi, Napoli, Italy; ^2^ Chirurgia Generale VII, Seconda Università degli Studi, Napoli, Italy

**Keywords:** ureteral stent, late complications, urinary stones

## Abstract

Encrustation constitutes a serious complication of ureteral stent use and can result in difficult stent removal. We report the case of a patient with a retained ureteral stent for 3 years following a radical cystectomy.

A 60-year-old man presented with history of recurrent antibiotic-resistant fever, sovrapubic pain, and urinary retention. Three years before, he had been submitted to radical cystectomy and neobladder reconstruction after the diagnosis of muscle-invasive bladder cancer. We do not have any information on the subsequent follow-up period.

A KUB plain film showed a huge urinary neobladder stone and other minor ones in the left ureter, associated with an indwelling ureteral double pigtail stent (Fig. 1). A CT scan was also performed and it confirmed this finding (Fig. 2). Due to the stone dimensions and after a discussion with the patient, it was decided to perform an open surgical procedure in order to remove the stones and the "forgotten" stent (Fig. 3). The patient was discharged without complication after 3 days.

Ureteral stents have proven to be an invaluable tool for urologists. Morbidity is minimal for up to 3 months, but longer indwelling times are associated with an increasing frequency of incrustation, infections, secondary stone formation, and obstruction of the stented tract[1]. Late complications of ureteral stents are frequent and appear in one-third of the patients[2]. Retained ureteral stents, especially those that are encrusted and associated with a stone burden, can be a difficult management problem[3]. Close follow-up of stented patients is valuable in early detection of morbidity or complications, and in such cases, the stent should be removed or exchanged as soon as possible.

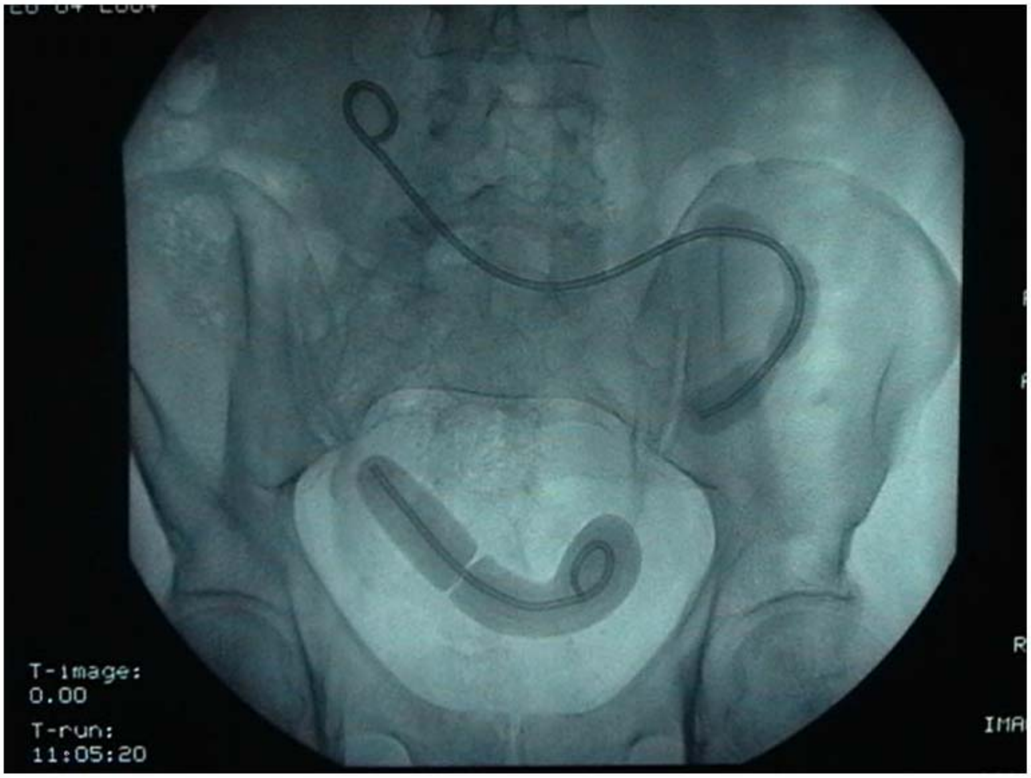

**FIGURE 1**. KUB plain film.

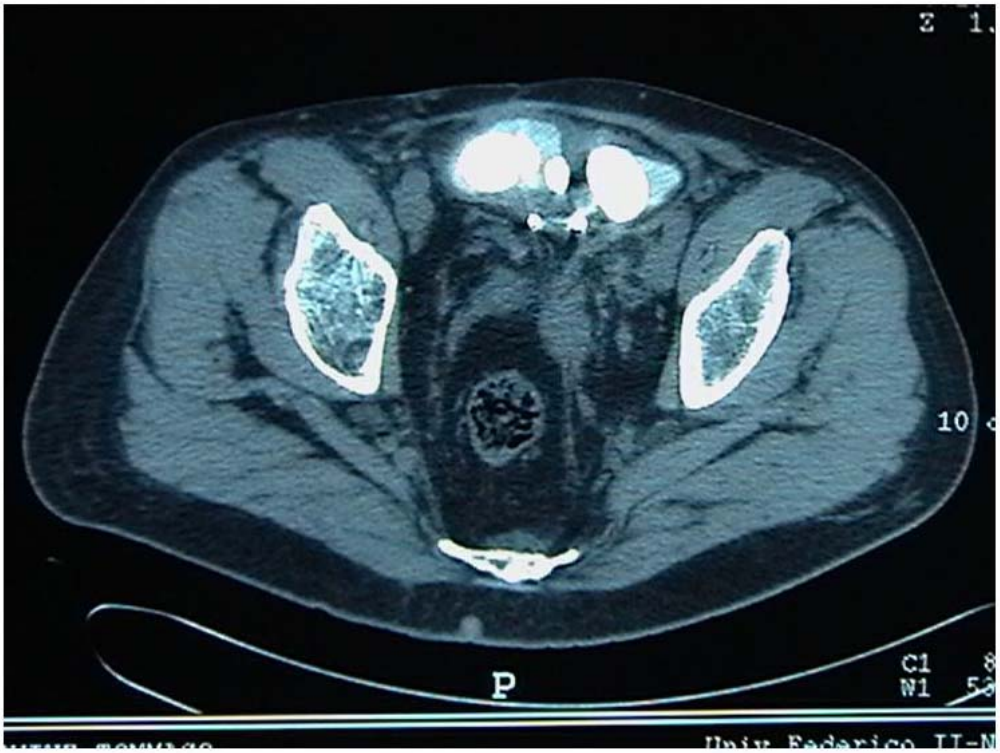

**FIGURE 2**. CT scan.

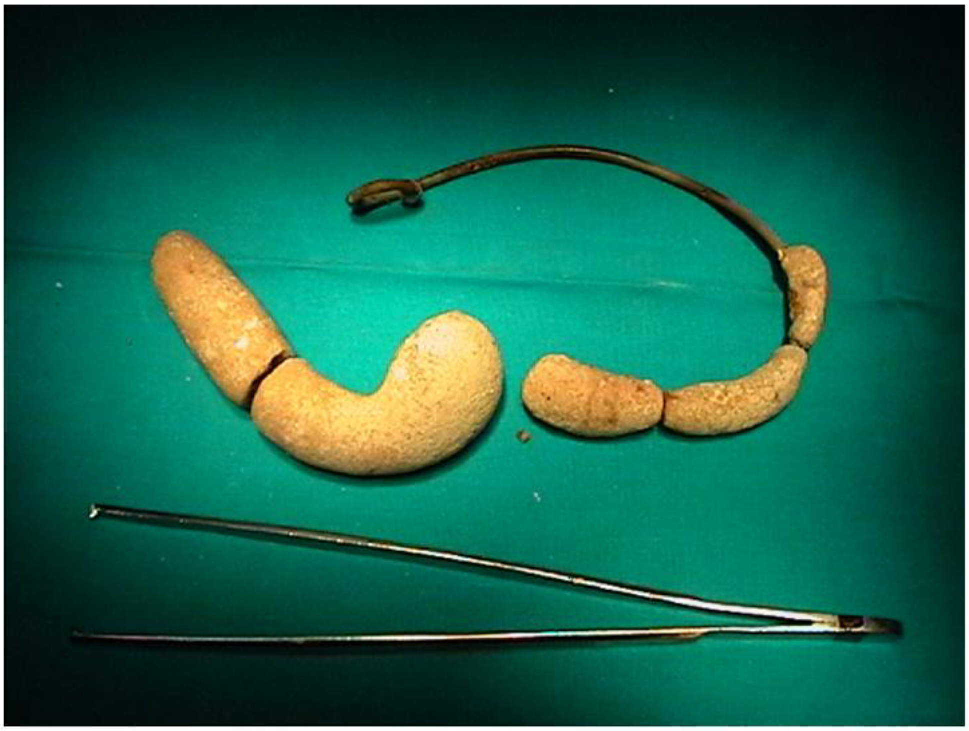

**FIGURE 3**. The removed stones and the forgotten stent.
